# The Effect of Acupressure on Sleep Quality in Menopausal Women: A Randomized Control Trial

**Published:** 2015-07

**Authors:** Zahra Abedian, Leila Eskandari, Hamid Abdi, Saeed Ebrahimzadeh

**Affiliations:** 1 Evidence-Based Care Research Centre, Department of Midwifery, School of Nursing and Midwifery, Mashhad University of Medical Sciences, Mashhad, Iran;; 2 Graduate of Midwifery, Health Center, Semnan University of Medical Science, Semnan, Iran;; 3 Specialist of Chinese Traditional Medicine, Dr. Sheikh Hospital, Mashhad University of Medical Sciences, Mashhad, Iran;; 4 Graduate Biostatistics, School of Nursing and Midwifery, Mashhad University of Medical Sciences, Mashhad, Iran

**Keywords:** Menopause, Sleep disorder, Acupressure

## Abstract

**Background:**

One of the common problems in menopausal women is sleep disorder. Traditional Chinese acupressure is a noninvasive and safe technique. Menopausal women can easily learn the technique and a self-care method to manage their sleep disorder. This study was carried out to evaluate the effectiveness of acupressure on sleep quality of postmenopausal women in Mashhad during 2009.

**Methods:**

This double blind, randomized clinical trial was performed on 120 qualified menopausal women at the age of 41-65 years. Their sleep quality was measured according to the Pittsburgh Sleep Quality Index (PSQI). Participants were randomly assigned to an acupressure group (n=37), a sham acupressure group (n=36) and a control group (n=32) by two time randomized method (systematic and simple randomized). These interventions were carried out for four consecutive weeks. The participants in the acupressure and sham acupressure groups learned to carry out the acupressure technique as a self-care at home with simultaneous massage techniques that were to be performed 2 hours before sleep, whereas only conversation was used in the control group. The data were analyzed by the SPSS software version 17.

**Results:**

The results indicated significant differences in total PSQI scores among the three groups (P<0.001). Tukey’s test revealed that there were significant differences between the acupressure group and the control group (P<0.001), the acupressure group and sham acupressure group (P<0.001), and the sham acupressure and the control group (P<0.001).

**Conclusion:**

Acupressure can be used as a complementary treatment to relieve sleep disorders in menopausal women; and is offered as an efficient method to manage sleep quality.

**Trial Registration Number: **IRCT2013100614910N1.

## Introduction

Menopause, the result of the cessation of ovarian function, is a natural phenomenon and a physiological process in middle-aged women.^1^^,^^2^ The menopausal women are in a more desirable position in comparison with the premenopausal period in terms of thoughts, experience and knowledge.^3^ Menopause is the most important midlife crisis among the menopausal females, as a result of specific physical and mental changes, which are mostly associated with low estrogen levels.^4^

The most common menopausal complications are hot flushes that can also lead to sleep disorders by night attacks and sweats.^5^ Sleep disorder may occur at any age, so that 35-40% of people experience sleep problems during the 5^th^ year.^6^ According to research reports, women have shown more problems such as inadequate or poor sleep, lack of sleep and light sleep.^7-9^ Sleep is one of the most important elements of human lives^10^ and improves the physical and mental abilities.^11^ On the other hand, poor sleep quality causes depression; irritability, poor concentration, and in the long run, it may be responsible for hypertension, cardiovascular diseases, diabetes, obesity and finally accelerates the aging process.^12^^,^^13^ Moreover, it can affect the social activities and one’s daily positive functioning.^14^

Some various drug and non-drug treatments are used to improve the sleep disorder.^15^ The most important reason for the limitation of drug treatment is their unwanted side effects.^16^ Nowadays, a large number of methods in non-drug treatments is proposed as alternative medicines.^17^ Acupressure is a form of Traditional Chinese Medicine (TCM) and a component of alternative treatments.^18^ It is effective in reducing pain, pregnancy nausea and sleep disorder in chronic patients and the elderly. The belief is that acupressure can help sleep disorders by changing the melatonin serum level and the secretion serotonin.^19^

Chen (1999) and Hosseinabadi (2008) evaluated the effectiveness of acupressure on sleep quality in the elderly; they reported a significant improvement in sleep quality in the acupressure group in comparison with both the control and placebo groups.^20^^,^^21^ In a study by Tsay et al., sleep quality in patients with dialysis significantly improved compared with the control group, but there were no differences between the sham and control groups.^22^ However, the results of Hisghman’s study did not support the efficacy of a standardized auricular acupoint prescription in adults with insomnia to significantly influence sleep efficiency.^16^ Acupressure has the potential to be learnt, can be taught to patients as a self-care method, and be easily used at home. It also reduces the dependency of patients to physicians.^23^

Women spend more than 1/3 of their lives in menopause, so they should be supported by providing menopause’s health information and promoting self-care behaviors. Considering the fact that there has been no research published on the effect of acupressure on the quality of menopausal women’s sleep, the present study is carried out to determine the general objective of the effect of acupressure on the menopausal females’ quality of sleep as integrated in managing women’s sleep disorder.

To our knowledge, there is no information on the effect of acupressure on sleep quality in menopausal women in Iran. Therefore, this study was conducted to respond to questions such as “would acupressure improve the sleep quality in menopausal women as a complementary treatment?” 

## Materials and Methods

**Figure 1:**
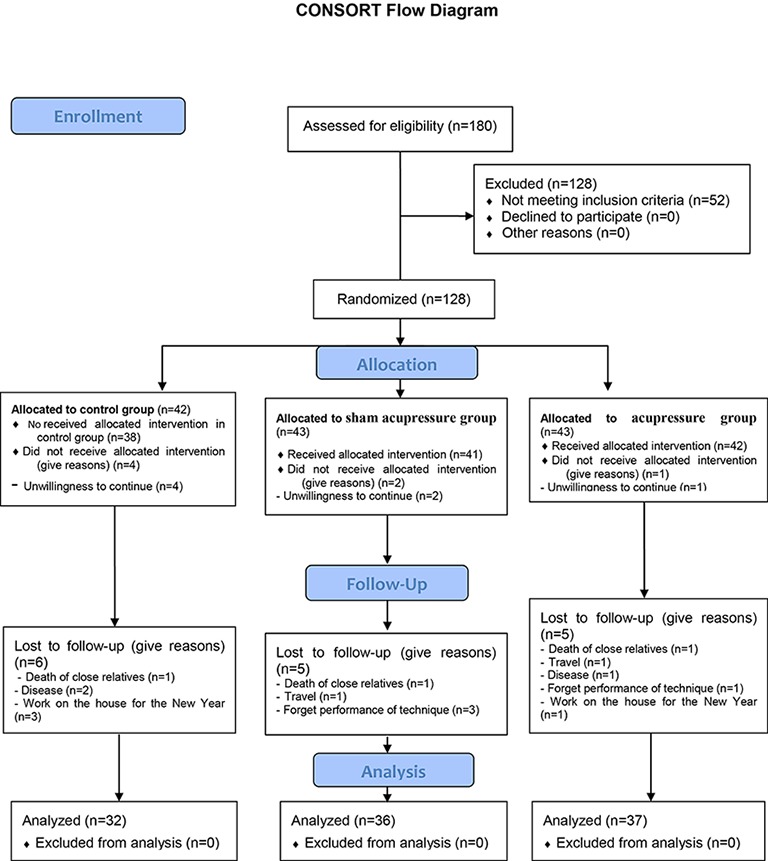
A diagram showing the sampling from the initiation through the follow-up of the trial.

This study was conducted as a 3-group, double blind (i.e. lack of knowledge helps the researcher and participant), and clinical trial to better understand the efficacy of acupressure on sleep quality in menopausal women. A total of 128 qualified menopausal women, 41-65 years of age who lived in Mashhad were selected. Of 180 menopausal women approached for participation, 52 did not meet the inclusion criteria as shown in the flow diagram (figure 1).

The sample size was determined based on the findings from a pilot study. It was calculated by using the mean comparison formula with a power of 80 percent and the correlation coefficient of 95 percent. The sampling method was done in two stages: initially the samples were selected by using a systematic method and then they were randomly assigned to three groups (table of random numbers): acupressure (n=37), sham acupressure (n=36) and control groups (n=32).

The inclusion criteria were: spending at least 12 months after amenorrhea or Follicle Stimulating Hormone (FSH) serum level ≥40 (1 year menopause), healthy pressure points, having full consciousness, having an acceptable ability to listen and speak the received training, the ability to perform self-care techniques at home, no mental and physical disorders, PSQI score higher than 5, and the ability to sit for about 30 minutes. 

The exclusion criteria were: emotional stress during the last 6 months, having specific eating diets, addiction to narcotic drugs, using drugs causing sleep disorders, suffering from special disease, taking hormonal drugs during the last 3-month, using herbal medicine to improve menopausal symptoms, body mass index (BMI) higher than 30, having moderate and severe depression based on Beck Depression Inventory, feeling of severe tiredness/fatigue and anxiety, circular job in particular night shifts, and lack of cooperation with researchers. 

After obtaining informed consent, the participants completed demographic information questionnaire, dedicated questionnaire to determine personal and environmental factors affecting sleep, Pittsburgh Sleep Quality Index (PSQI), and a checklist to record the night sleep pattern.

The participants in the acupressure group were asked to massage the effective pressure points and the participants in the sham acupressure group were asked to massage the sham pressure points as a self-care method at home.

Four acupoints were chosen for the individuals in the acupressure group, namely Shenmen on the wrist crease, Sanyinjiao point (SP6) on both feet, Fengchi on the hairline of the back neck (occipital area) and Yintang, at the top of the nose on the centerline between the ends of the eyebrows. 

Non-acupoints, which were 1 to 3 CUN (Chinese unit of length) away from the true acupoints, were used in the sham acupressure group. These points were out of the energy pathway.

At the end of the training, as a reminder, the researcher marked the points of the sham acupressure and acupressure medicine on the points of Sanyinjiao and Shenmen. The precision of acupressure was confirmed if the subjects felt fully pressured.

Interventions were carried out for 4 weeks with the same time length and technique in both the acupressure and non-acupressure groups. The intervention time was limited to 10 minutes. It was done 1 to 2 hours before sleeping, each night (except Fridays) by circular massage covering 1 cm diameter. Participants were trained regarding the force of finger pressure. They were required to feel the full pressure of approximately 3-4 kg on the points without pain.

The control group only received the weekly control of blood pressure and speech communication about health question. 

During the study period, participants in the three groups were asked to fill out the checklist of the night sleep pattern to record the duration of their sleep period. Both intervention groups had to determine the time of performing their daily massage technique.

Content validity was used to confirm the validity of questionnaires. PSQI reliability was confirmed by the internal consistency method using Alpha Chronbach (ᾱ =0.84).

The SPSS software version 17 was used to register and analyze the data. The one-way ANOVA and Kruskal-Wallis were used to compare continuous numerical variables. For categorical variables, the chi square, Fisher exact and Tukey tests were used. 

## Results

**Figure 2:**
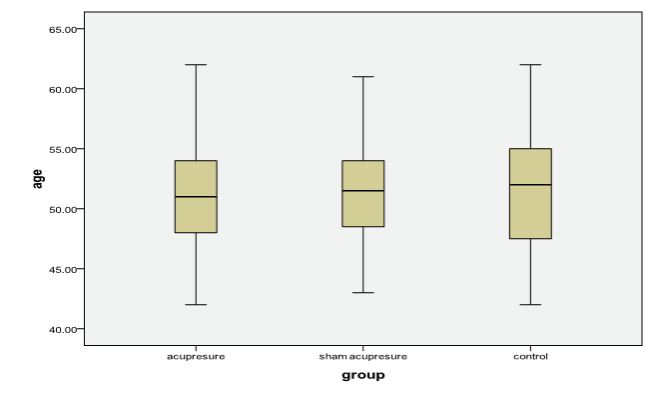
The mean age of subjects is compared between the three study groups.

There were no significant differences between the mean age (years) of participants in the acupressure group (50.7±4.7), sham acupressure group (51.3±4.2), and the control group (51.4±5.5) (P=0.894) (figure 2).

The mean duration of menopause in the acupressure group was 49±49 months, in the sham acupressure group was 46±36 months, and in the control group was 48±41 months. There were no significant differences between them (P=0.895). 

All participants were homemakers. The results of Kruskal-Wallis and Fisher exact test showed no significant differences between the three groups in terms of: the level of education (P=0.584), the level of education of father(P=0.323), exercise times per week (P=0.826), daily nap duration (P=0.897), time of sleeping (P=0.516), lamp lightening (P=0.87) and noise in the bedroom (P=0.9), the condition of bed (P=0.914), hot flash at night (P=0.464), sweating while sleeping (P=0.336), family income (P=0.563), physical activities (P=0.826), exposure to fresh air and sunshine (P=0.97), housing condition (P=0.284), and using special methods for getting a better sleep (P=0.980). 

At the beginning of the study, the results of ANOVA showed that there was no significant difference between the three groups in terms of the total mean score and PSQI elements (table 1).

**Table 1:** The pre-test comparison of sleep quality between acupressure, sham acupressure and control groups

**Quality of sleep** **(elements of PSQI)**	**Acupressure group** **(n=37)**	**Sham acupressure group** **(n=36)**	**Control group** **(n=32)**	**P value**
	**Mean** **±** **SD**	**Mean** **±** **SD**	**Mean** **±** **SD**	
Subjective sleep quality	1.83±0.76	1.80±0.88	1.78±0.79	0.930*
Sleep latency	2.37±0.75	2.33±0.88	2.65±0.65	0.162*
Sleep duration	2.13±0.71	2.19±0.82	2.09±0.73	0.810*
Habitual sleep efficiency	1.54±0.90	1.52±0.87	1.68±0.84	0.647*
Sleep disturbance	2.02±0.60	1.08±0.50	1.93±0.66	0.693*
Sleeping medication	0.32±0.74	0.72±1.23	0.37±0.60	0.575*
Daily performance	1.97±0.76	2.00±0.82	2.15±0.75	0.723*
Total PSQI	12.13±3.06	12.66±3.80	12.66±3.31	0.745**

*One-way ANOVA; **Kruskal-Wallis

Based on the post-test results by paired t-test, there was a significant difference in the total score and the whole elements of PSQI (except sleep medication) in the acupressure group (P≤0.001). In groups with sham acupressure points, a significant statistical difference was observed, at the end of the study, in the scores of subjective sleep quality (P≤0.001), sleep latency (P≤0.003), sleep duration (P≤0.014), sleep disturbance (P≤0.002) and the PSQI total score (P≤0.001). In the control group, there was no significant difference in any of the elements and the total PSQI scores (table 2). However, there was a significant difference between the three groups (P≤0.001). Post hoc comparison pointed out that there was a significant difference between both the acupressure (P≤0.001) and the sham acupressure groups (P≤0.001) with the control group. In addition, the difference between the acupressure and sham acupressure groups was significant (P≤0.001).

**Table 2:** The mean differences of pre-test and post-test of sleep quality among acupressure, sham acupressure and control groups

**Quality of sleep** **(elements of PSQI)**	**Acupressure group** **(n=37)**		**Sham acupressure group** **(n=36)**		**Control group** **(n=32)**	
	**Mean** **±** **SD**	**P value**	**Mean** **±** **SD**	**P value**	**Mean** **±** **SD**	**P value**
Subjective sleep quality	0.97±0.64	0.001	0.64±0.83	0.001	0.10±0.68	0.447
Sleep latency	1.52±0.86	0.001	0.50±0.94	0.003	0.09±0.68	0.447
Sleep duration	0.78±0.62	0.001	0.38±0.90	0.014	0.03±0.64	0.786
Habitual sleep efficiency	0.56±0.68	0.001	0.27±1.03	0.115	0.18±0.85	0.226
Sleep disturbance	0.75±0.79	0.001	0.36±0.63	0.002	0.03±0.59	0.768
Sleeping medication	0.13±0.48	0.001	0.01±0.88	0.001	0.15±0.98	0.325
Daily performance	0.97±0.64	0.001	0.19±0.98	0.242	0.12±0.81	0.521
Total PSQI	5.08±1.29	0.001	2.19±3.63	0.001	0.21±2.39	0.609

To assess improvement in sleep quality, the rate of change from the beginning to the end of the study was descriptive (table 3).

**Table 3:** Descriptive of percent improvement in sleep quality of the acupressure, sham acupressure and control groups

**Quality of sleep** **(elements of PSQI)**	**Percent improvement (%)**		
	**Acupressure group** **(intervention effect)**	**Sham acupressure group** **(mental effect)**	**Control group** **(time effect)**
Subjective sleep quality	53.2	35.0	5.6
Sleep latency	43.0	22.0	3.5
Sleep duration	36.0	17.0	10.0
Habitual sleep efficiency	37.0	18.0	2.0
Sleep disturbance	37.1	33.0	-4.5
Sleeping medication	4.0	1.3	-5.0
Daily performance	49.0	9.7	2.0
Total PSQI	41.0	17.0	2.0

## Discussion

In the present study, two groups of sham acupressure point and control groups were used in order to prevent the effect of sham acupressure point as well as the group of acupressure points. The acupressure group was used to survey the effect of pressure on the acupressure points, which are effective in sleep quality. Additionally, through the sham acupressure groups, they can survey the mental effect due to pressure on the non-effective point and the time factor through the control group.

The result of this study showed that sleep quality improved in the acupressure group of women with menopause. These findings are consistent with the studies by Hosseinabadi,^21^ Tsay,^22^ Chen,^20^ and Sun^24^ who examined the effect of acupressure on promoting the sleep quality in other populations.

Previous studies have confirmed that acupressure techniques release neurotransmitters such as serotonin and activate opioid systems. Therefore, by producing body relaxation, it can improve sleep quality.^20^

Although sleep quality improved in the sham acupressure group, there were significant improvements in the total PSQI score of the acupressure group in comparison with the sham acupressure group. The total improvement of PSQI in comparison with the baseline was 41% in the acupressure group and 17% in the sham acupressure group. Such difference in improvements could be mainly caused by the effects of massage on Fingchi and Chenmen that led to enhancement of sleep quality. 

The reason for sleep quality improvement in the sham acupressure group seems to be the same massage techniques in two groups of real and non-real acupoints. Massaging points, especially on the area of shoulder, neck, or head itself may produce various levels of body relaxation and thus produces higher sleep quality. In addition, it can be due to the mental and emotional effects of massage. 

The present study differs from previous studies. Sampling of the past research has been based on the objective and it was done on a limited centers such as elderly institutions or dialysis centers. In addition, the researchers used the acupressure medicine on themselves. However, in our study, the cases in the intervention groups were trained on the acupressure points, to be carried out at home as a self-care method.

Since acupressure is non-invasive and cheap with a short learning curve, patients can easily learn it as a self-care method. It allows minimal patient’s dependence on the healthcare system, reduces the number of visits to outpatients, free up scarce hospital resources, reducing costs to the healthcare system, and finally improves patient’s health and satisfaction.^25^

In this study, the total improvement score in the control group was 2%, which improved over time. Also in the sham acupressure group, we gained 17% improvement in sleep quality, in which 2% was related to time factor and 15% attributed to the psychological effects of massage in the sham acupressure points.

In the case of the acupressure group, 41% improvement was observed. Deducing the component of the psychological effects of massage (17%) and time factor (15%), the attributed effect of the true acupressure points on sleep quality was 22%.

## Conclusion

It is concluded that the acupressure alone can improve sleep quality at a rate of 22% in menopausal women by massage on the effective points. Researchers suggest that acupressure may have an important role in managing sleep disturbances and improve sleep quality in women with menopause. It can be used as a self-care method based on complementary treatment for such sleep disorders. 
